# Supplementary material for: Electrochemical on-surface synthesis of a strong electron-donating graphene nanoribbon catalyst
Source: Nat Commun. 2024 Jul 29;15:5972. doi: 10.1038/s41467-024-50086-6 (PMC11286955; doi:10.1038/s41467-024-50086-6)
Supplement: Supplementary file 1 — Supplementary Information [file 41467_2024_50086_MOESM1_ESM.pdf]

Supplementary Information

Electrochemical on-surface synthesis of a strong electron-donating graphene nanoribbon catalyst

Hiroshi Sakaguchi[1]*, Takahiro Kojima[1], Yingbo Cheng[1], Shunpei Nobusue[1] and Kazuhiro Fukami[2]

Correspondence to: sakaguchi@iae.kyoto-u.ac.jp

Contents

Materials and Methods 3
Synthesis 3
2-Butoxynaphthalene 3
Preparation of Au (111) substrate 4
Preparation of iodine-covered Au (111) 4
CVD synthesis of 5-AGNR and 7-AGNR 4
CV measurement 4
Raman spectroscopy 4
Calculations 4
Bandgap simulation 4
Raman simulation 5
LDOS mapping simulation 5
Supplementary figures 6
Supplementary References 26

Materials and Methods

Synthesis

A JEOL JNM-ECP300 (300 MHz for ^{1}H) instrument was used. NMR spectra were measured in parts per million using tetramethylsilane (δ 0.00 ppm for chloroform-*d*) as the internal standard for ^{1}H NMR. Thin-layer chromatography (TLC) and column chromatography were performed on Art. 5554 (Merck KGaA) and silica gel 60N (Kanto Chemical Co.), respectively. Gel permeation chromatography (GPC) was performed on an LC-9201 system (Japan Analytical Industry) with JAIGEL 1H and 2H polystyrene columns (eluent: $CHCl_3$, flow: 10 mL/min).

2-Butoxynaphthalene

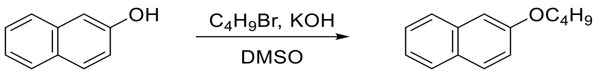

A solution of 2-naphthol (2.88 g, 20.0 mmol), 1-bromobutane (3.30 g, 24.1 mmol) and KOH (2.25 g, 40.1 mmol) in DMSO (30 mL) was stirred at 95°C overnight. The reaction mixture was diluted with water and cooled in a refrigerator. The precipitated solid was separated by filtration and purified by chromatography (silica gel, hexanes) followed by GPC ($CHCl_3$) to afford 2-butoxynaphthalene (2.88 g, 14.4 mmol, 72% yield) as a white solid. ^{1}H NMR (300 MHz, $CDCl_3$) δ 7.77-7.70 (m, 3H), 7.42 (ddd, J = 6.9, 6.9, 1.2 Hz, 1H), 7.32 (ddd, J = 6.9, 6.9, 1.2 Hz, 1H), 7.31 (s, 1H), 7.14 (dd, J = 6.9, 1.2 Hz, 1H), 4.08 (t, J = 6.6 Hz, 2H), 1.88-1.79 (m, 2H), 1.60-1.48 (m, 2H), 1.00 (t, J = 7.2 Hz, 3H).

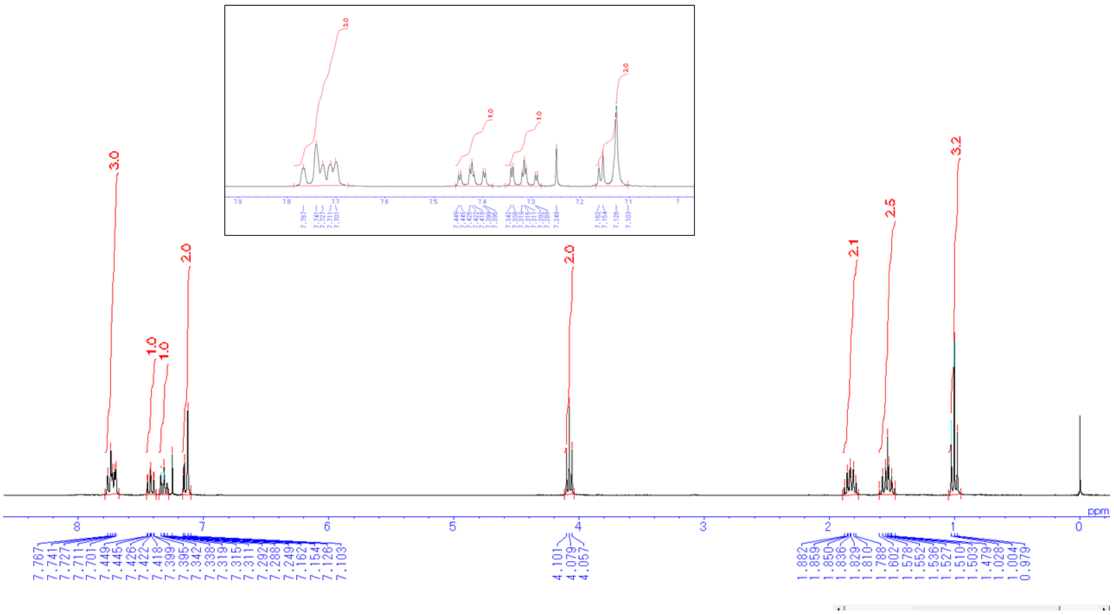

Preparation of Au (111) substrate

An Au (111) substrate for electrochemical GNR growth was made using a homemade e-beam vacuum-deposition system. Evaporated gold was deposited on 2.8 × 2.8 cm^2 mica or 1.0 × 1.3 cm^2 glass substrates, which were heated at 350°C under a vacuum of 2.6 × 10^{-6} Pa. A layer up to 40 nm was deposited at a rate of 0.5 Å s^{-1}.

Preparation of iodine-covered Au (111)

An Au (111)/mica substrate was immersed in 10 mM iodine/chloroform solution for 2 min. The substrate was then washed with chloroform and dried on a hot plate at 70°C for 5 min.

CVD synthesis of 5-AGNR and 7-AGNR

The two-zone CVD system used a quartz tube (26 mm*ϕ*, 86 cm) as the reactor, with a rotary pump that could evacuate the system to below 7 × 10^{-4} Torr. There was also a two-zone electric furnace with a temperature controller, an Ar gas flow system with a mass-flow controller, and a mantle heater for evaporating precursors. To synthesize GNRs, the precursor (30 µg) was placed in a quartz boat, with the Au(111)-deposited mica or glass substrate, inside the quartz tube. Ar was introduced into the tube at a flow rate of 500 sccm, resulting in a vacuum of 1 Torr. Synthesis of GNRs occurred in two stages. In the first stage, the temperature of zone 2 in the quartz tube was set to 250°C, resulting in evaporation of the precursors. The path of precursors through zone 1 was heated to 350°C. In the second stage, the temperature of zone 2 was increased to convert polymers into GNRs by dehydrogenation. Specific synthetic conditions for 5-AGNR and 7-AGNR are found in reference[1].

CV measurement

All CV measurements were conducted using a Solartron Analytical SI 1287 electrochemical workstation with a scan rate of 50 mV s^{-1}. Platinum and silver wires served as the counter and reference electrodes, respectively. The electrolyte solution consisted of 100 mM tetrabutylammonium hexafluorophosphate in *o*-DCB. Cell fabrication was performed in a glove box. An Au (111) was used as the working electrode for CV measurements of the precursor solution (Supplementary Fig. 4). A 5 mM solution was used for TTF CV measurement (Fig. 2h). For the CV measurement of electrochemically produced GNR, 5-AGNR and 7-AGNR, all GNRs were formed on Au (111) substrates (Fig. 2h and Supplementary Fig. 11).

Raman spectroscopy

Raman spectroscopy was performed using a Raman instrument (Lambda Vision Inc, LVRAM500/532) with a 532-nm excitation laser.

Calculations

Bandgap simulation

Bandgap simulations were conducted through the CASTEP module[2] of the BIOVIA Materials Studio software suite (Dassault Systèmes SE). Molecular mechanics (MM)

were utilized to optimize the structure before performing Density Functional Theory (DFT) calculations. To carry out the MM calculation, the COMPASS II force field with the Forcite module was used. For DFT structural optimization and bandgap simulations, the CASTEP module was utilized. The exchange-correlation functional used was the Perdew-Burke-Ernzerhof generalized-gradient approximation (GGA-PBE)[3], and Ultrasoft pseudopotentials were generated on-the-fly (OTFG).

Raman simulation

Raman simulations were conducted using the CASTEP module in the BIOVIA Materials Studio software suite (Dassault Systèmes SE). Norm-conserving pseudopotentials were employed with 16 × 4 × 1 k-points. The plane-wave cutoff energy was set to 830 eV. The maximum threshold for force and displacement tolerances during structural optimization was 0.1 eV $Å^{-1}$ and 0.0005 Å, respectively. Methoxy groups were used in place of butoxy groups to reduce the computational cost.

LDOS mapping simulation

Local density of states (LDOS) calculations were obtained at the Perdew-Burke-Ernzerhof generalized-gradient approximation (GGA-PBE)[3] level as implemented in $DMol^3$ (BIOVIA Materials Studio software, Dassault Systèmes SE). 10-mer of GNR was used for the calculation. For the LDOS mapping simulation, a height of 2.8 Å was selected.

Supplementary figures

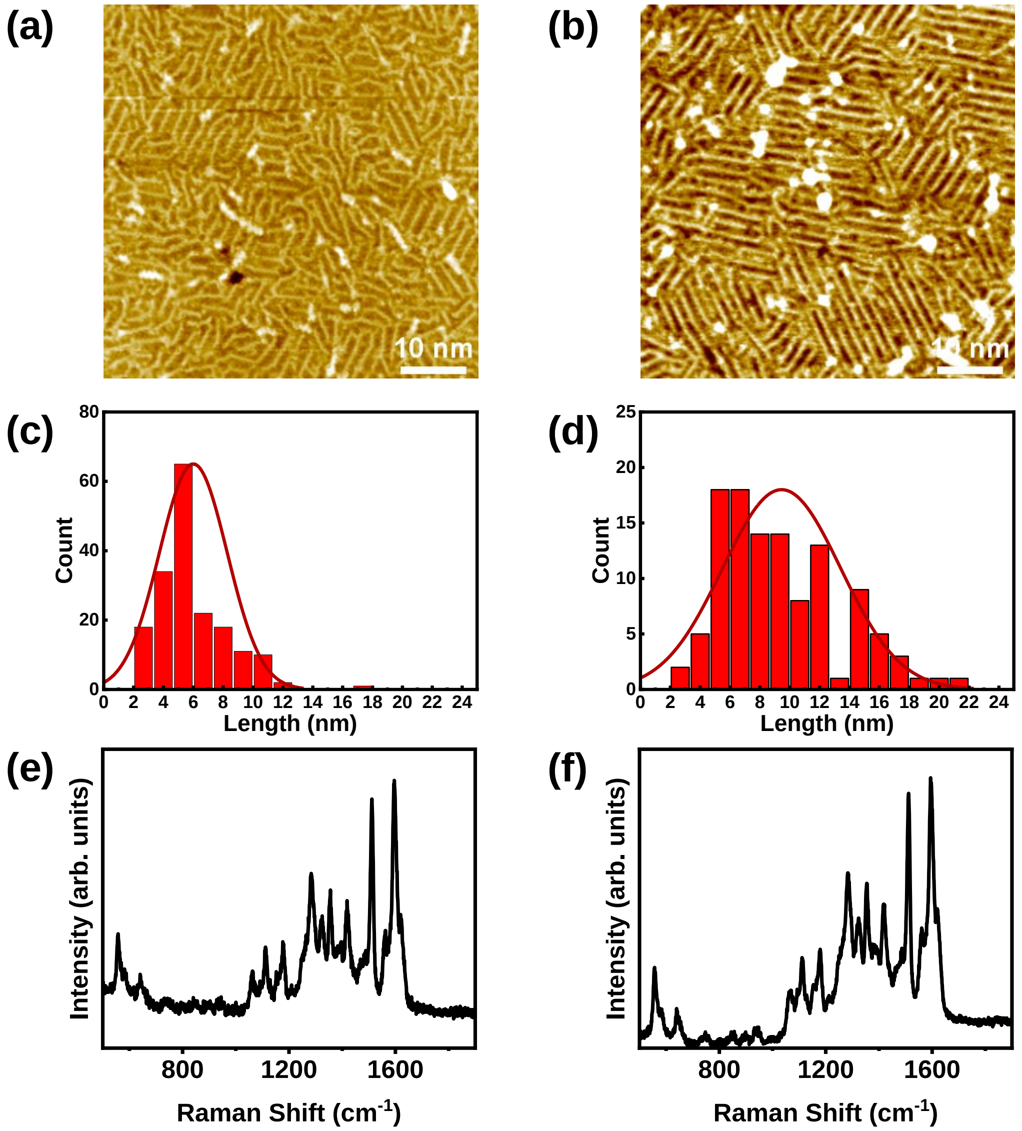

Supplementary Fig. 1. Ambient STM images (-0.2 V, 5 pA) of electrochemically produced GNR on (a) Au (111) and (b) iodine-covered Au (111). Histograms of strand lengths of electrochemically produced GNRs on (c) Au (111) and (d) iodine-covered Au (111). Raman spectra of electrochemically produced GNRs on (e) Au (111) and (f) iodine-covered Au (111).

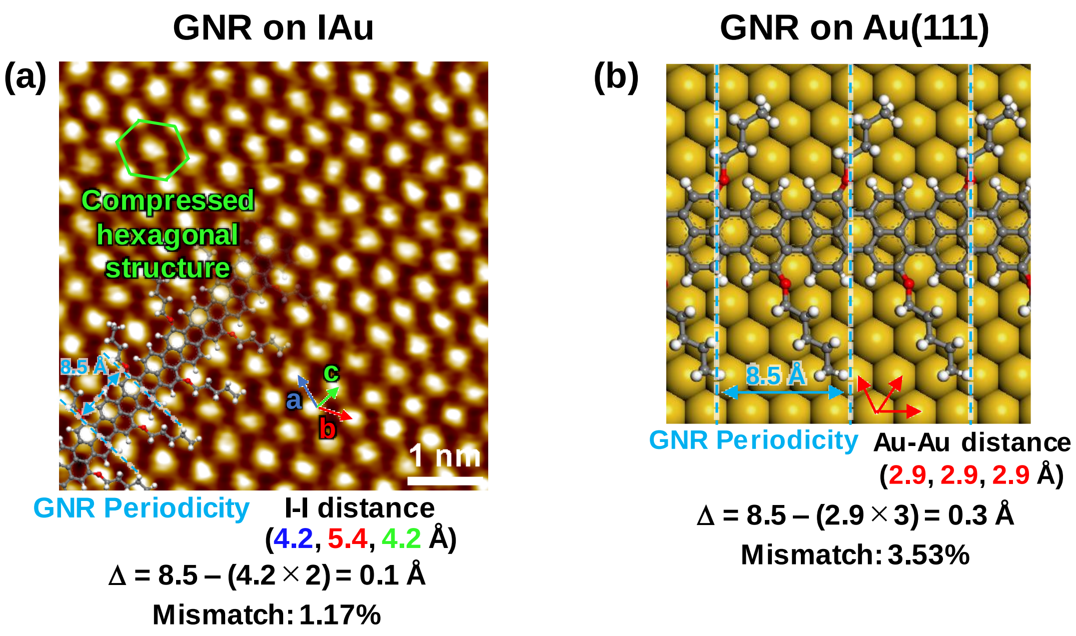

Supplementary Fig. 2. (a) LT-STM image of iodine-covered Au (111) with a superimposed structure of GNR. The inset shows a, b, and c axes, and a compressed hexagonal lattice structure. I-I distances along the a, b, and c axes are 4.2, 5.4, and 4.2 Å , respectively. The mismatch between GNR and substrate is 1.17%. (b) Lattice matching between the GNR and Au(111). Au-Au distances along the a, b, and c axes are 2.9, 2.9, 2.9 Å, respectively. The mismatch between GNR and substrate is 3.53%.

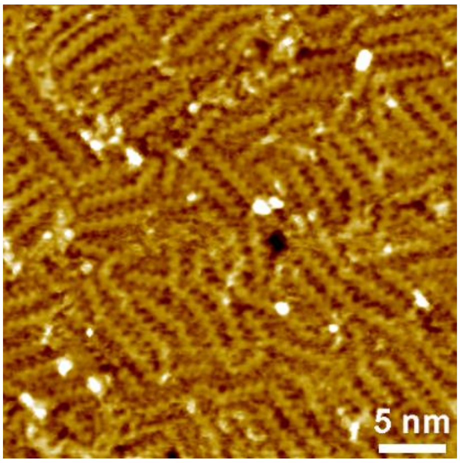

Supplementary Fig. 3. Ambient STM image of electrochemically produced GNR on iodine-covered Au(111) at room temperature (5 V, 0.5 sec, 30 cycles).

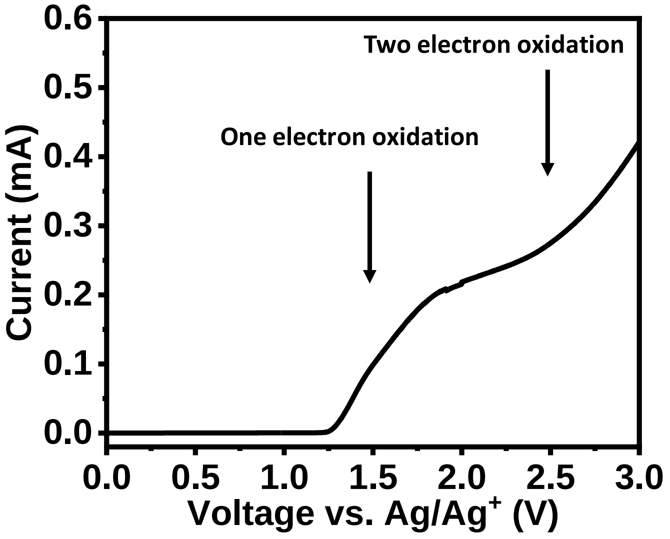

Supplementary Fig. 4. Cyclic voltammogram of 5 mM 2-butoxynaphthalene with 0.1 M $TBAPF_6$ in *o*-DCB. The scan rate was 50 mV s^{-1}.

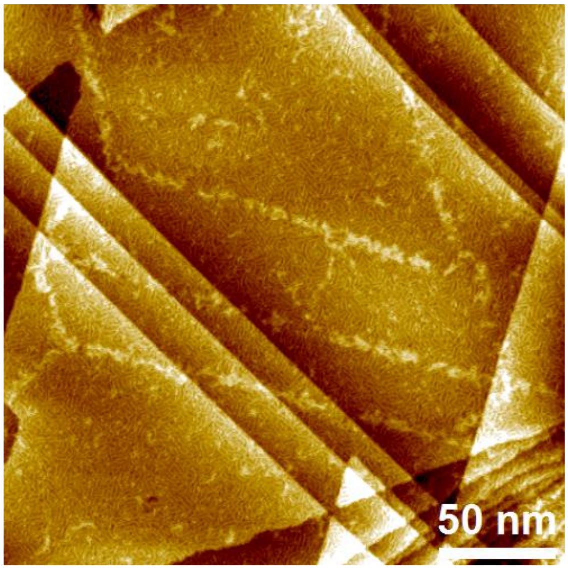

Supplementary Fig. 5. Large area LT-STM (-0.61 V, 580 pA) image of electrochemically produced GNR (200 $\times$ 200 nm).

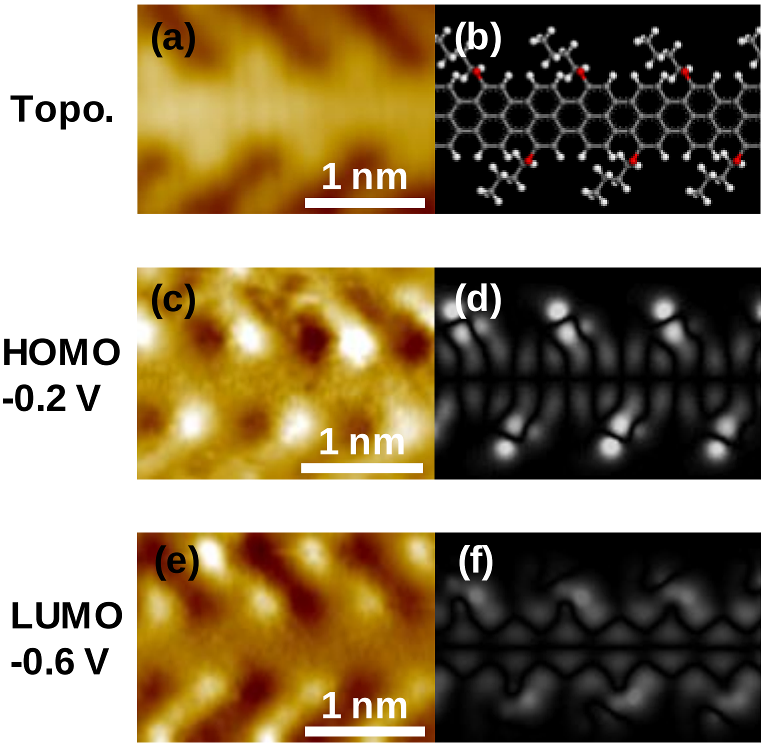

Supplementary Fig. 6. Experimental LT-STM topography of electrochemically produced GNR on iodine-covered Au(111) (a) and molecular models (b). Experimental constant-height d*I*/d*V* maps (c,e) and simulation of LDOS maps (d,f).

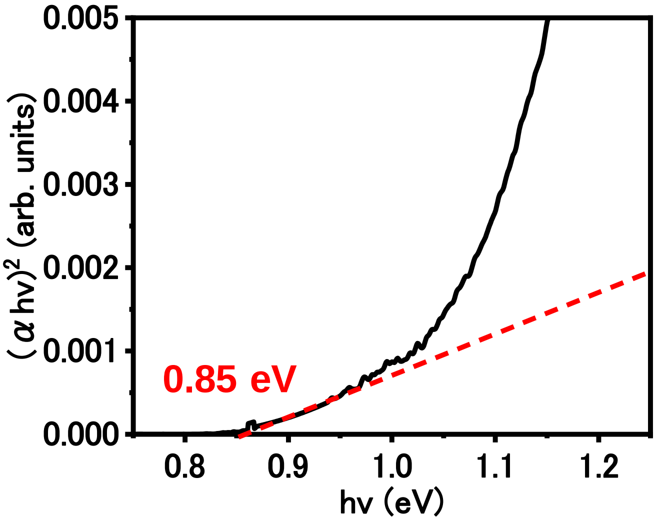

Supplementary Fig. 7. Tauc plot obtained from the optical absorption spectrum of electrochemically produced GNR film on an ITO electrode showing the bandgap value.

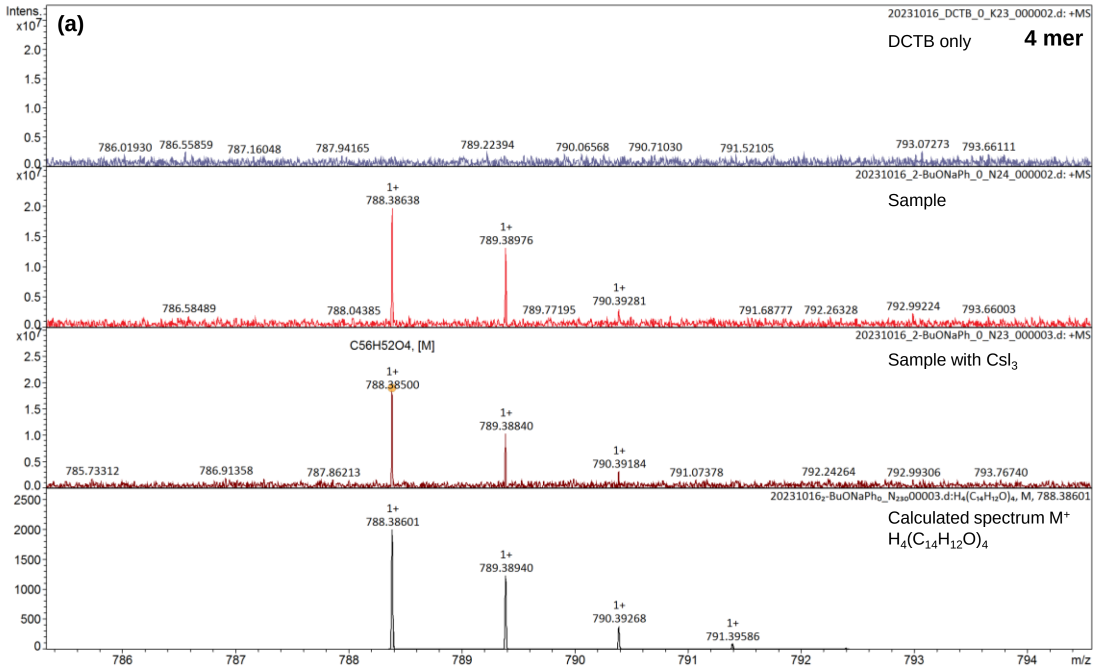
(a)
4 mer
Intens. x10^7
20231016_DCTB_0_K23_000002.d: +MS
DCTB only
786.01930 786.55859 787.16048 787.94165 789.22394 790.06568 790.71030 791.52105 793.07273 793.66111
20231016_2-BuONaPh_0_N24_000002.d: +MS
Sample
1+ 788.38638
1+ 789.38976
1+ 790.39281
786.58489 788.04385 789.77195 791.68777 792.26328 792.99224 793.66003
20231016_2-BuONaPh_0_N23_000003.d: +MS
Sample with CsI3
C56H52O4, [M]
1+ 788.38500
1+ 789.38840
1+ 790.39184
785.73312 786.91358 787.86213 791.07378 792.24264 792.99306 793.76740
Calculated spectrum M+
H4(C14H12O)4
1+ 788.38601
1+ 789.38940
1+ 790.39268
1+ 791.39586
m/z

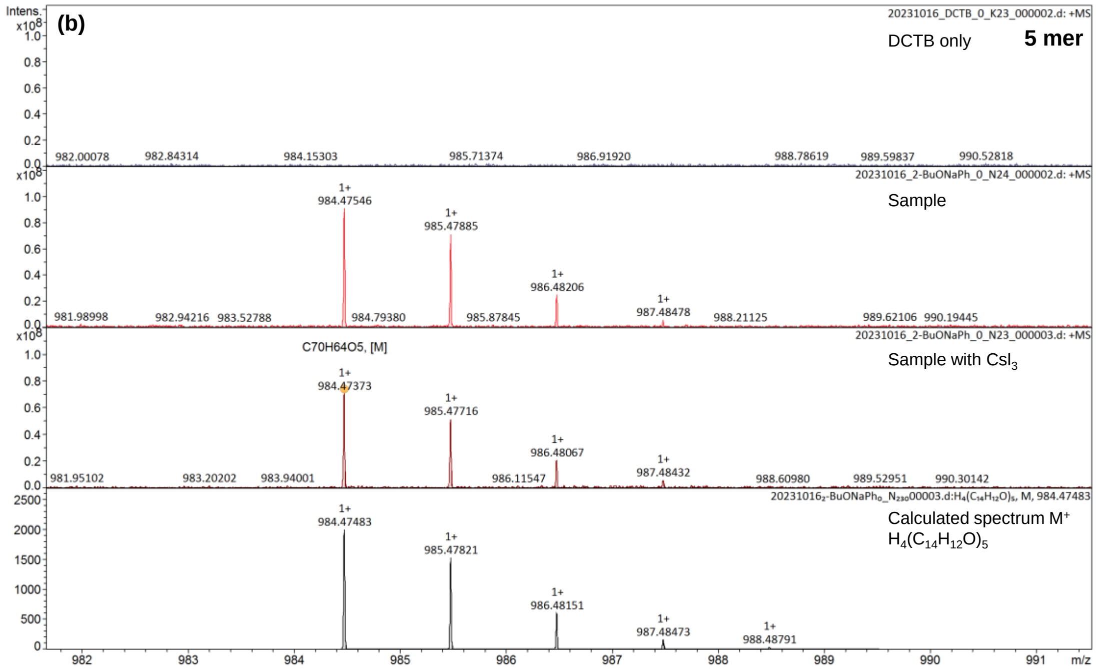
(b)
5 mer
Intens. x10^8
20231016_DCTB_0_K23_000002.d: +MS
DCTB only
982.00078 982.84314 984.15303 985.71374 986.91920 988.78619 989.59837 990.52818
20231016_2-BuONaPh_0_N24_000002.d: +MS
Sample
1+ 984.47546
1+ 985.47885
1+ 986.48206
1+ 987.48478
981.98998 982.94216 983.52788 984.79380 985.87845 988.21125 989.62106 990.19445
20231016_2-BuONaPh_0_N23_000003.d: +MS
Sample with CsI3
C70H64O5, [M]
1+ 984.47373
1+ 985.47716
1+ 986.48067
1+ 987.48432
981.95102 983.20202 983.94001 986.11547 988.60980 989.52951 990.30142
Calculated spectrum M+
H4(C14H12O)5
1+ 984.47483
1+ 985.47821
1+ 986.48151
1+ 987.48473
1+ 988.48791
m/z

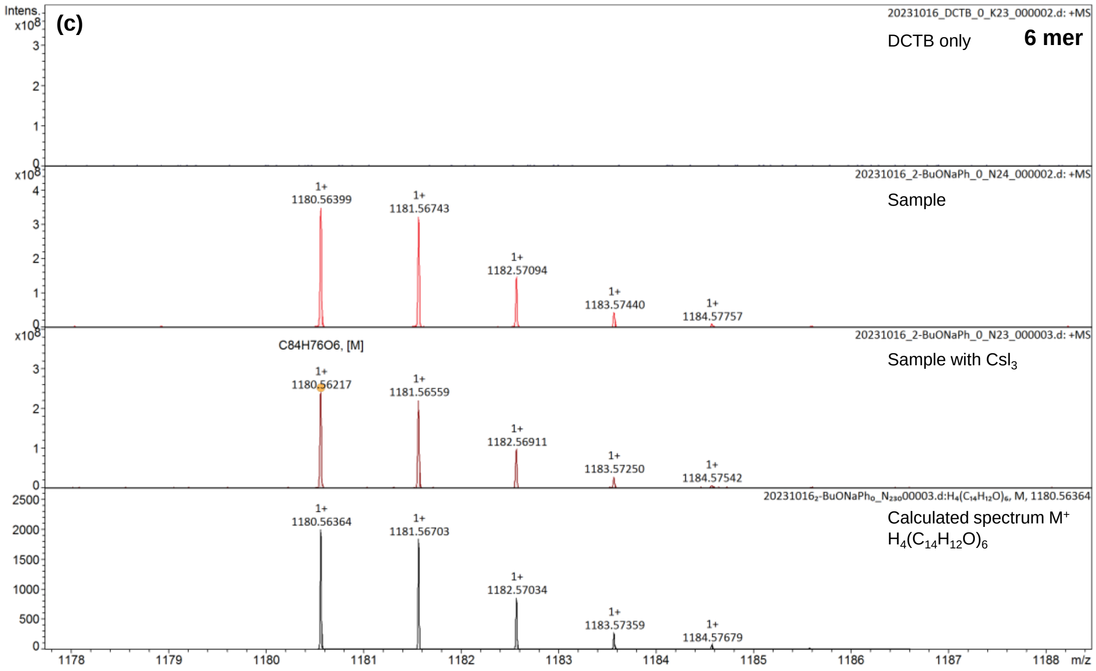
(c)
6 mer
DCTB only
Sample
Sample with CsI$_3$
Calculated spectrum M$^+$
$H_4(C_{14}H_{12}O)_6$
C84H76O6, [M]
1180.56399
1181.56743
1182.57094
1183.57440
1184.57757
1180.56217
1181.56559
1182.56911
1183.57250
1184.57542
1180.56364
1181.56703
1182.57034
1183.57359
1184.57679
m/z

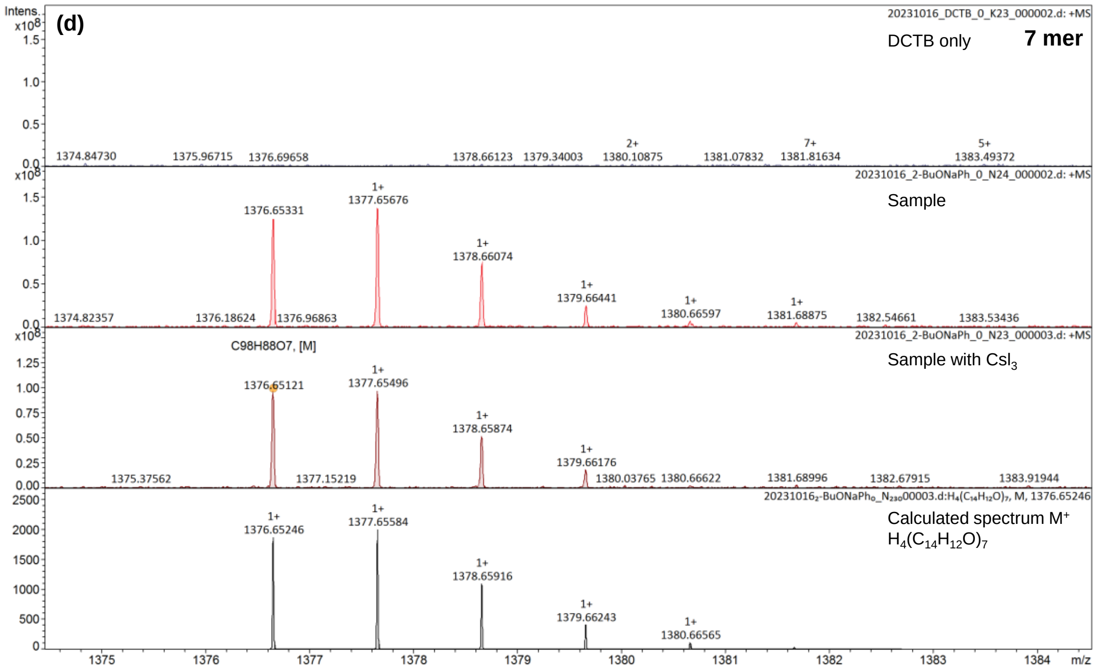
(d)
7 mer
DCTB only
Sample
Sample with CsI$_3$
Calculated spectrum M$^+$
$H_4(C_{14}H_{12}O)_7$
C98H88O7, [M]
1376.65331
1377.65676
1378.66074
1379.66441
1380.66597
1381.68875
1376.65121
1377.65496
1378.65874
1379.66176
1376.65246
1377.65584
1378.65916
1379.66243
1380.66565
m/z

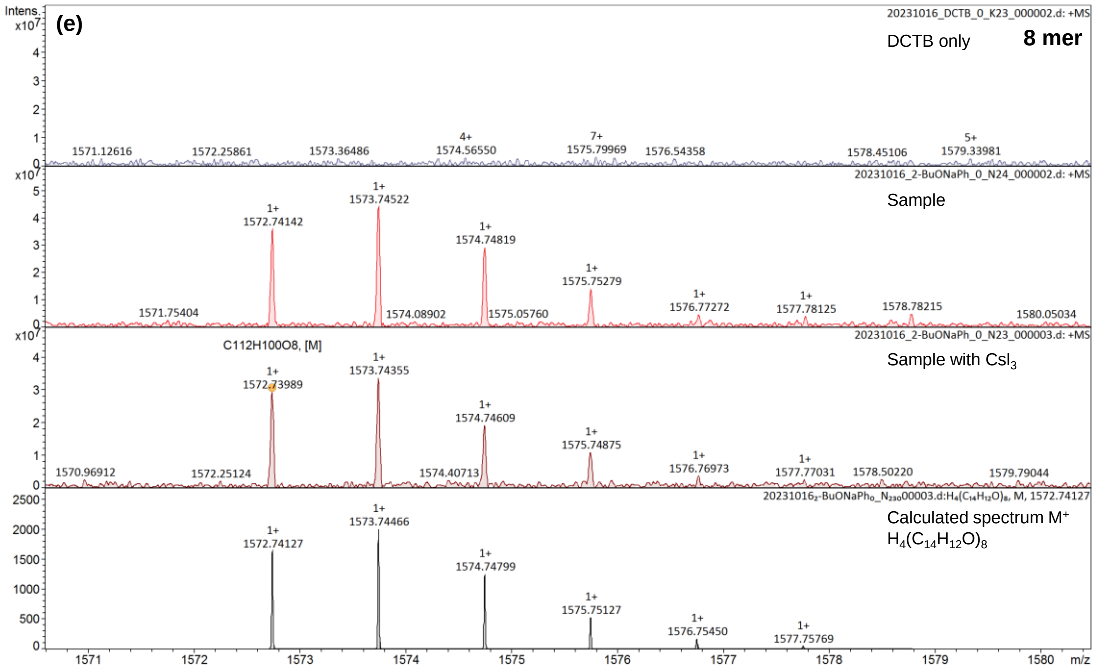

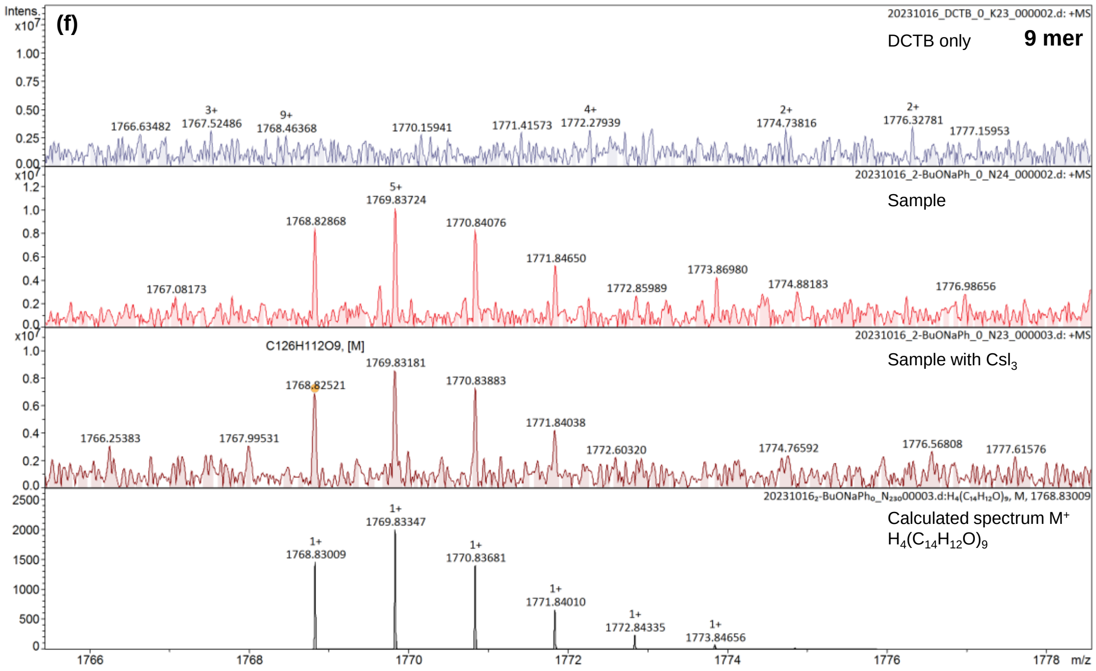

Supplementary Fig. 8. (a-f) MALDI-FT-ICR MS analysis of electrochemically produced GNR with unit length of 4, 5, 6, 7, 8, 9 mers, respectively. *trans*-2-[3-(4-*tert*-Butylphenyl)-2-methyl-2-propenylidene]malononitrile (DTCB) was used as a matrix.

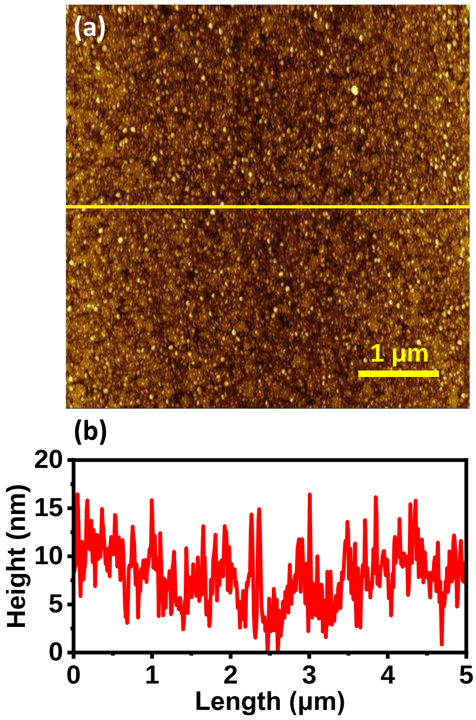

Supplementary Fig. 9. AFM image (a) and cross section (b) of the ITO substrate.

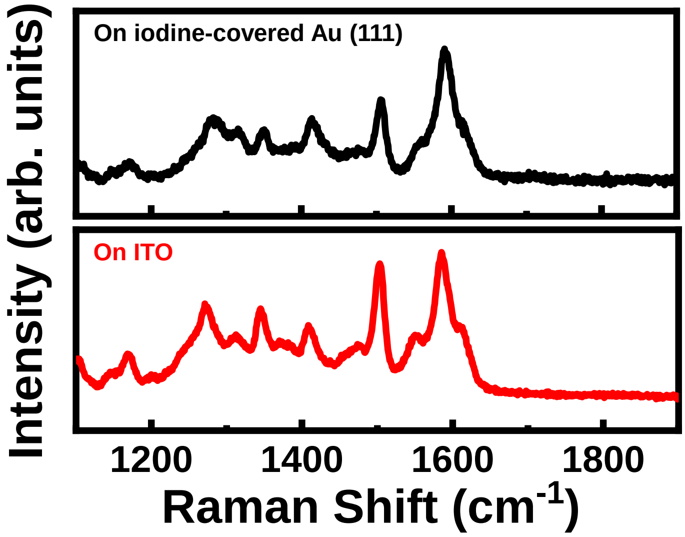

Supplementary Fig. 10. Raman spectra of electrochemically produced GNRs formed on iodine-covered Au(111) and ITO substrates (5 V, 0.5 sec, 30 cycles).

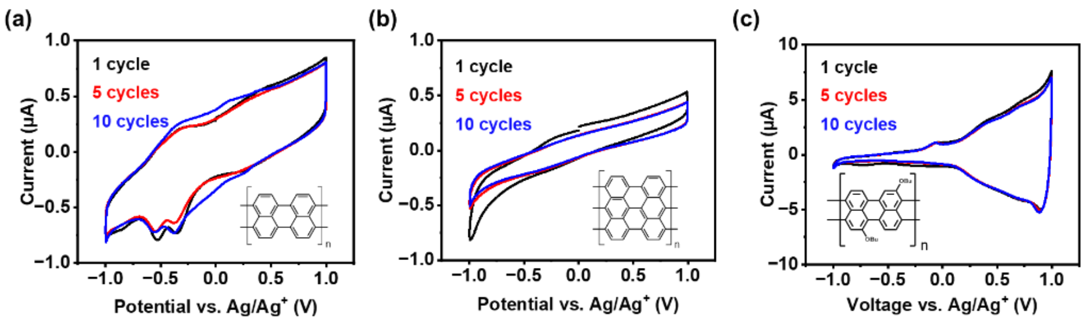

Supplementary Fig. 11. CV curves (scan rate of 50 mV s^{-1}) of (a) 5-AGNR, (b) 7-AGNR and (c) electrochemically produced GNR, deposited on Au (111) measured in electrolyte solution with 0.1 M $TBAPF_6$ in *o*-DCB. Black, red and blue curves show 1, 5 and 10 cycles, respectively. The inset shows the chemical structures.

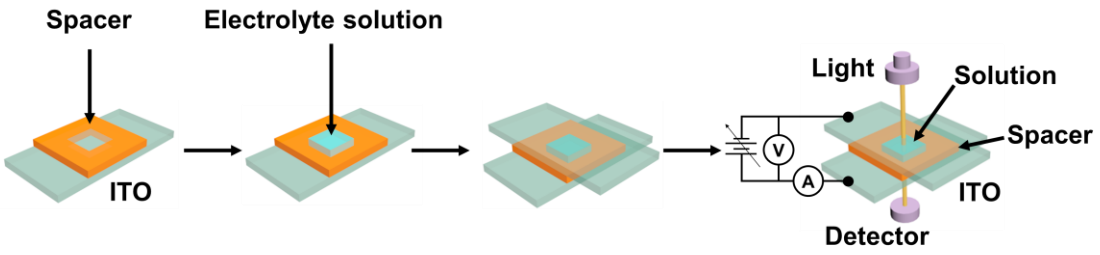

Supplementary Fig. 12. Fabrication process of the electro-absorption cell.

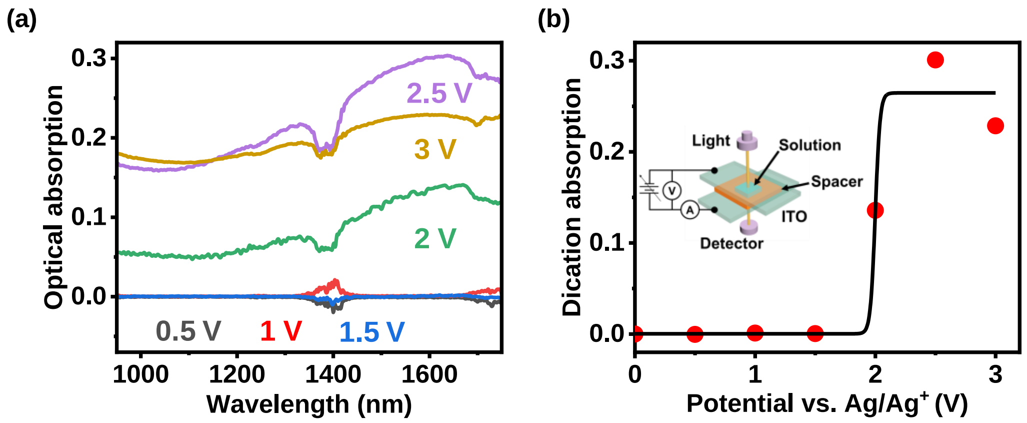

Supplementary Fig. 13. (a) Electro-absorption of the sample cell (5 mM 2-butoxynaphthalene in electrolyte solution) after applying voltages for 15 s. (b) Voltage dependence on absorption of dication in the sample cell observed at 1600 nm. The inset shows the experimental setup.

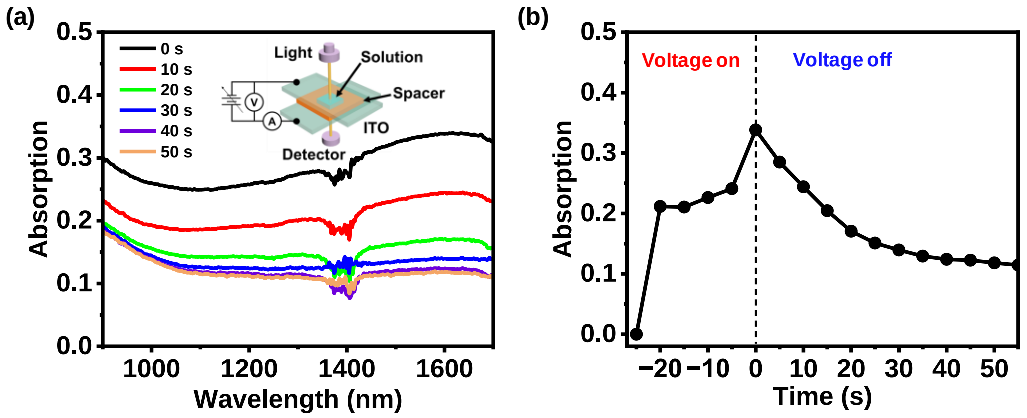

Supplementary Fig. 14. (a) Time-dependent electro-absorption spectra of the sample cell after 2.5 V application for 25 s. The inset shows the experimental setup. (b) Temporal profile of electroabsorption at 1600 nm.

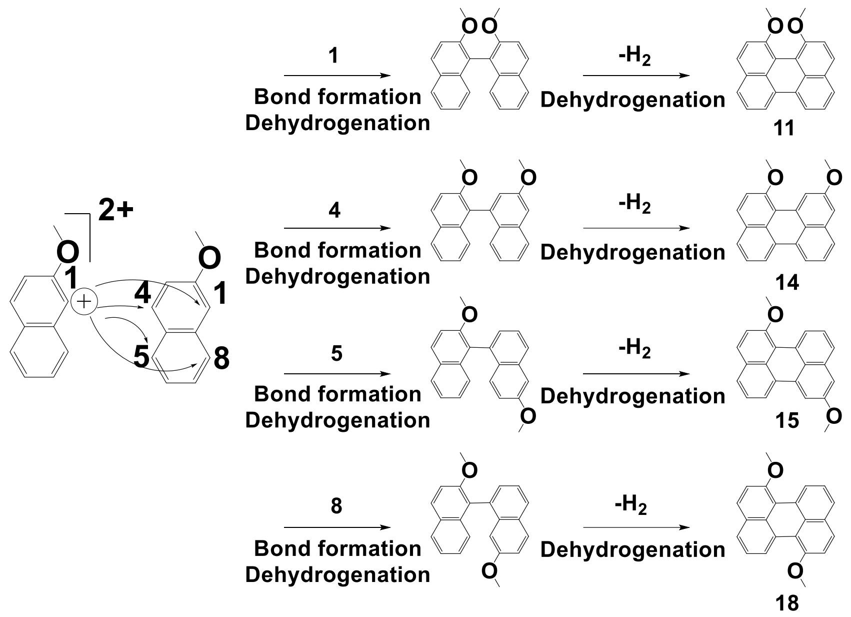

Supplementary Fig. 15. Possible reaction pathways and products of the dimer.

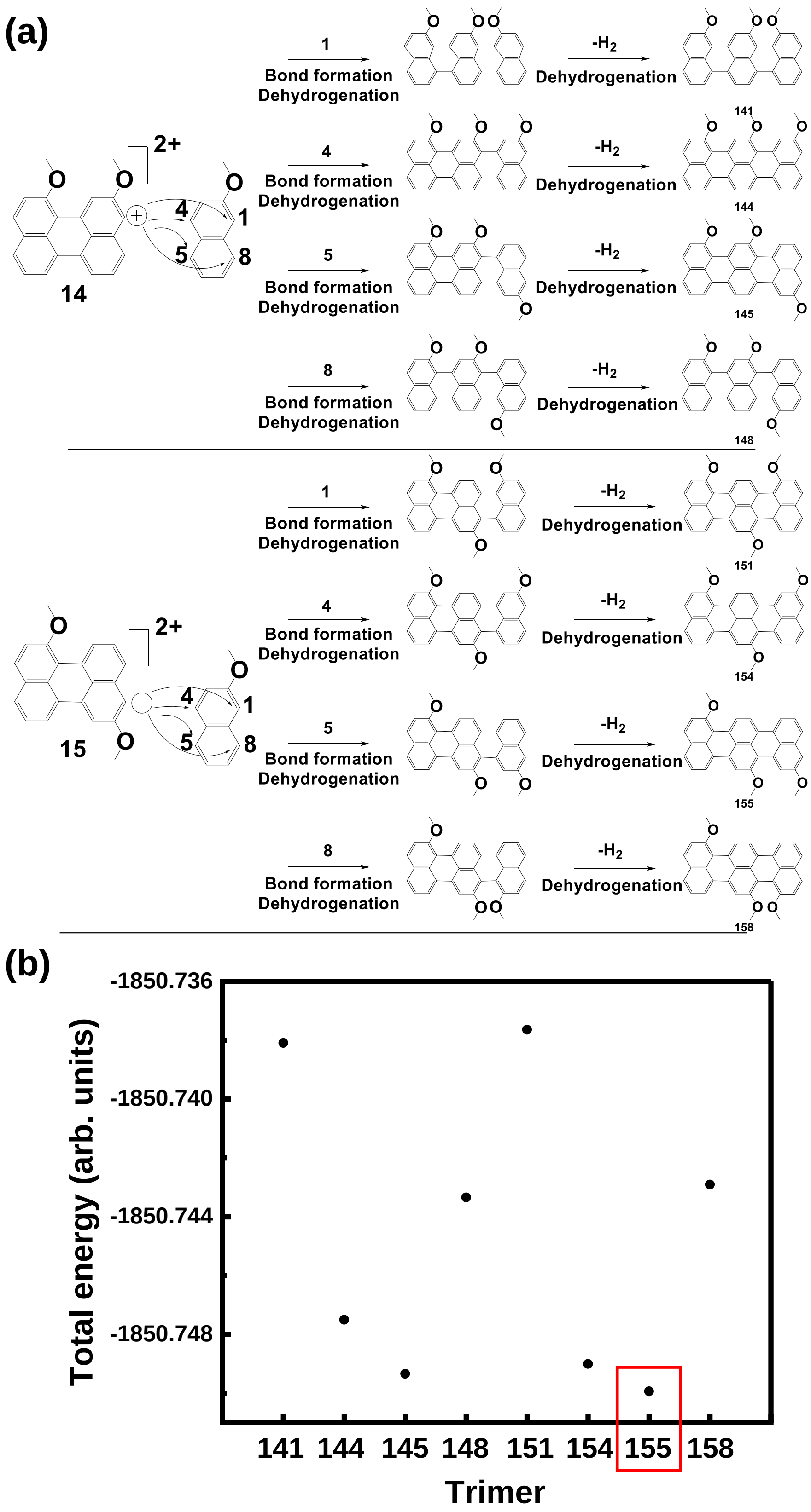

Supplementary Fig. 16. (a) Possible reaction pathways and products of the trimer. (b) Total energies of possible trimer products. The red square shows minimum energy.

(a)

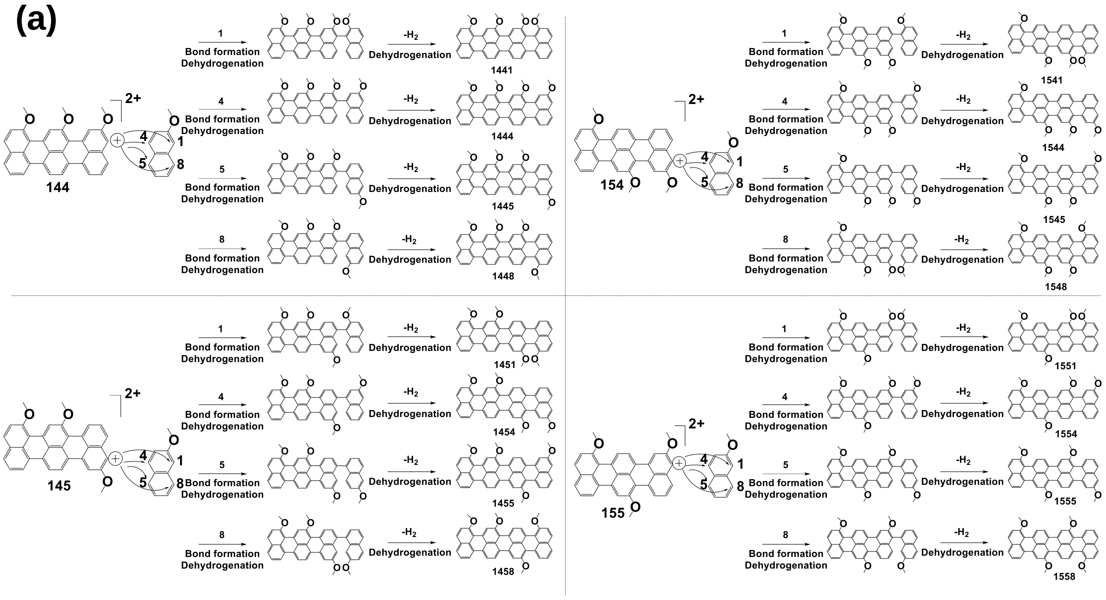

(b)

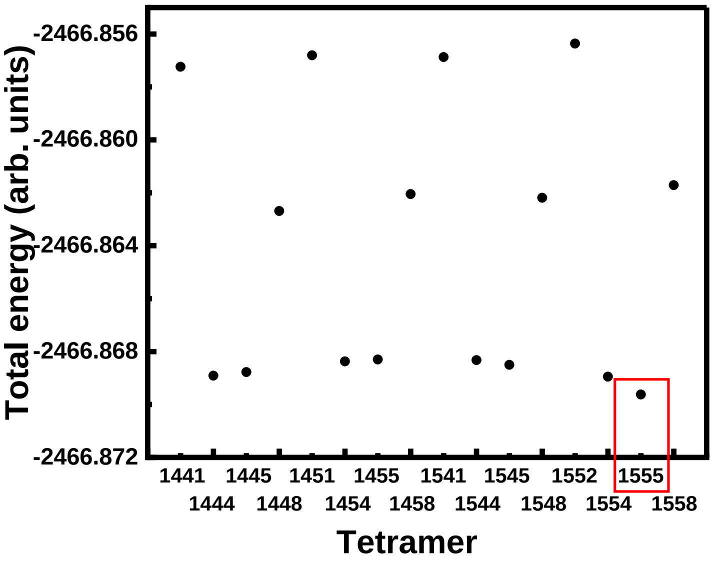

Supplementary Fig. 17. (a) Possible reaction pathways and products of the tetramer. (b) Total energies of possible tetramer products. The red square shows minimum energy.

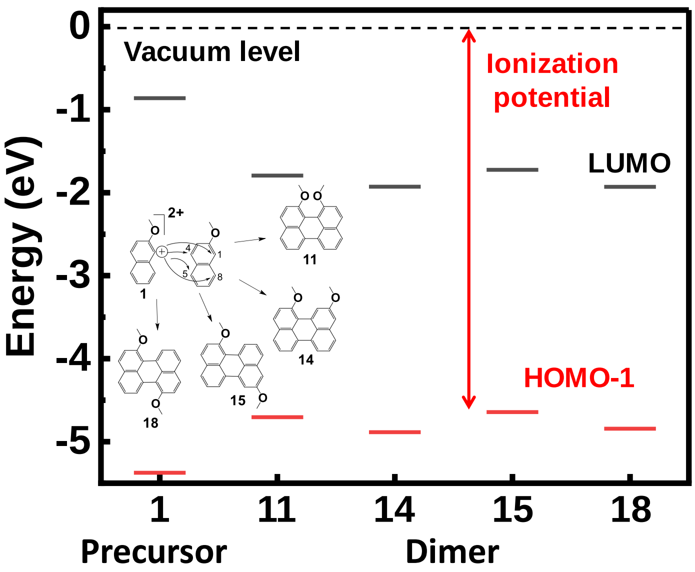

Supplementary Fig. 18. Energy levels of HOMO-1 and LUMO for possible dimer products and precursor.

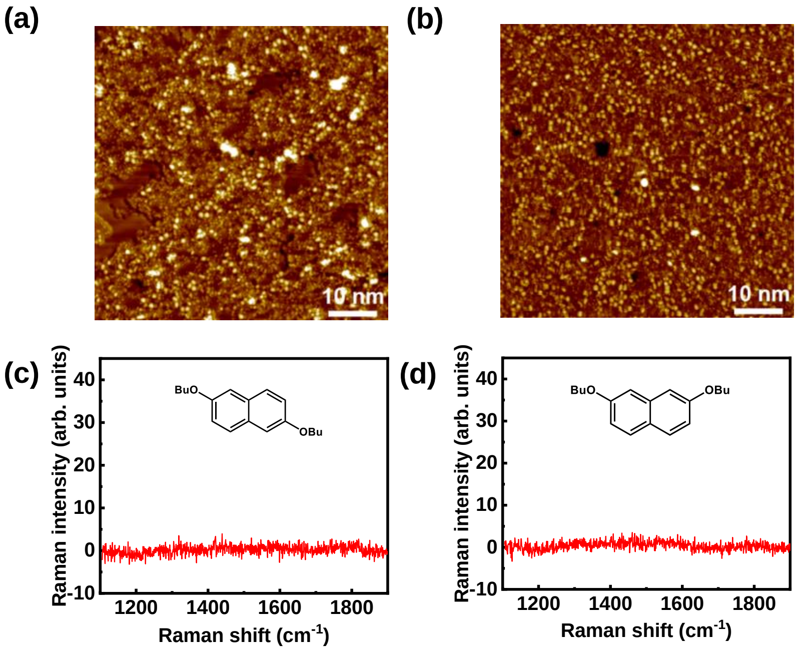

Supplementary Fig. 19. LT-STM (0.2 V, 5 pA) images and Raman spectra of electrochemically treated Au (111) in a 5 mM sample solution of (a, c) 2,6-dibutoxynaphthalene, (b, d) 2,7-dibutoxynaphthalene with 0.1 M of $TBAPF_6$ in *o*-DCB after voltage application (5 V, 0.5 s, 3 cycles), respectively. Panels of (c) and (d) show chemical structures.

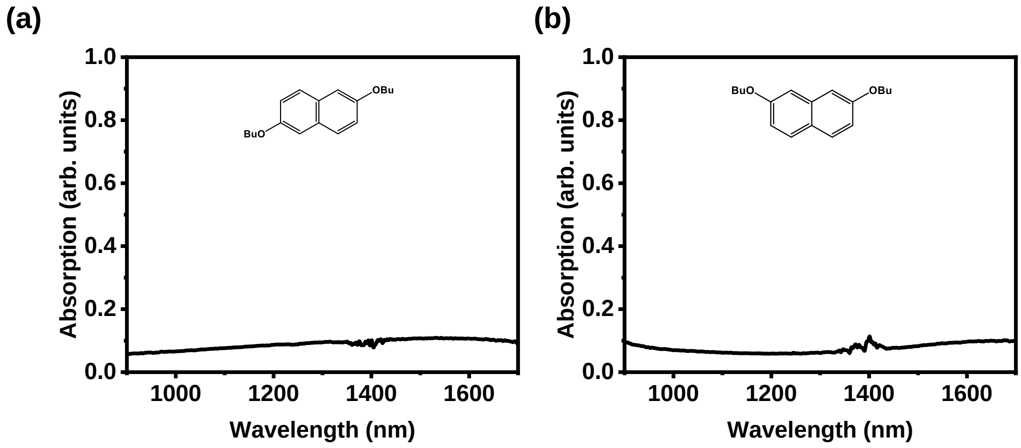

Supplementary Fig. 20. Electro-absorption spectra of the sample cell (5 mM in electrolyte solution) of (a) 2,6-dibutoxynaphthalene and (b) 2,7-dibutoxynaphthalene after 2.5 V application for 25 s. Panels (a) and (b) show chemical structures.

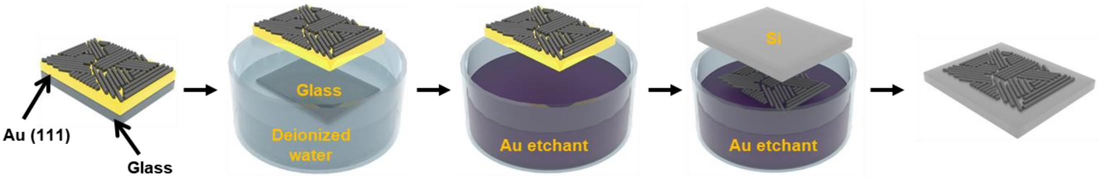

Supplementary Fig. 21. Illustration of the transfer process of electrochemically produced GNR from Au (111) to Si. 50 mM of I_2 and 1.1 M of KI aqueous solution was used as the Au etchant.

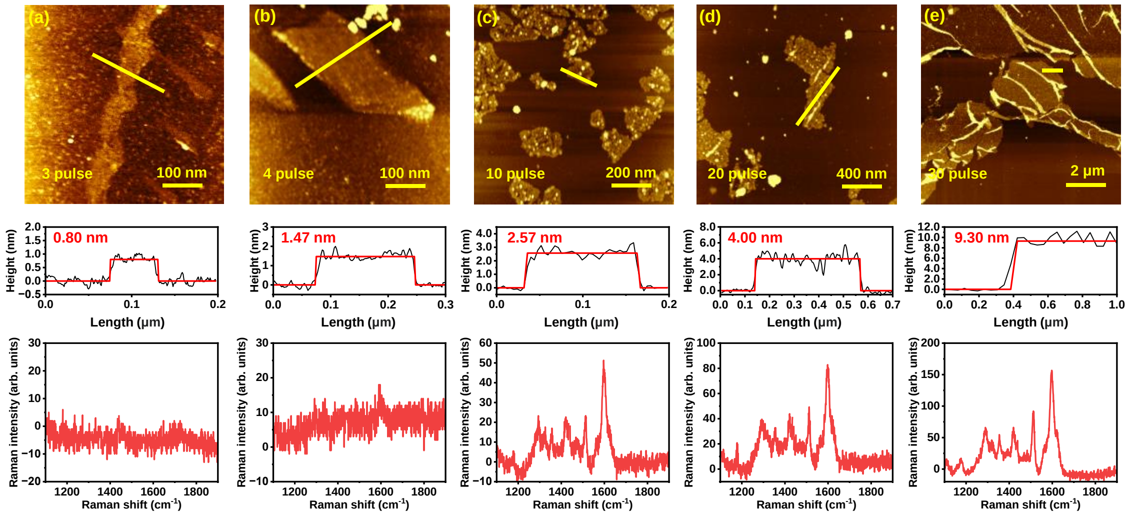

Supplementary Fig. 22. (a-e) AFM images, cross sections and Raman spectra of GNRs obtained after applying various numbers of voltage cycles (3, 4, 10, 20, and 30 cycles) (5 V, 0.5 sec).

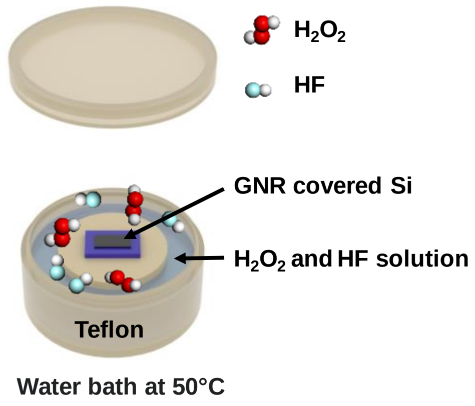

Supplementary Fig. 23. Illustration of the Si vapor-etching process. The etchant solution consisted of 25 mL of 46% HF and 0.5 mL of 30% H_2O_2. Etching proceeded at 50°C for 1 h.

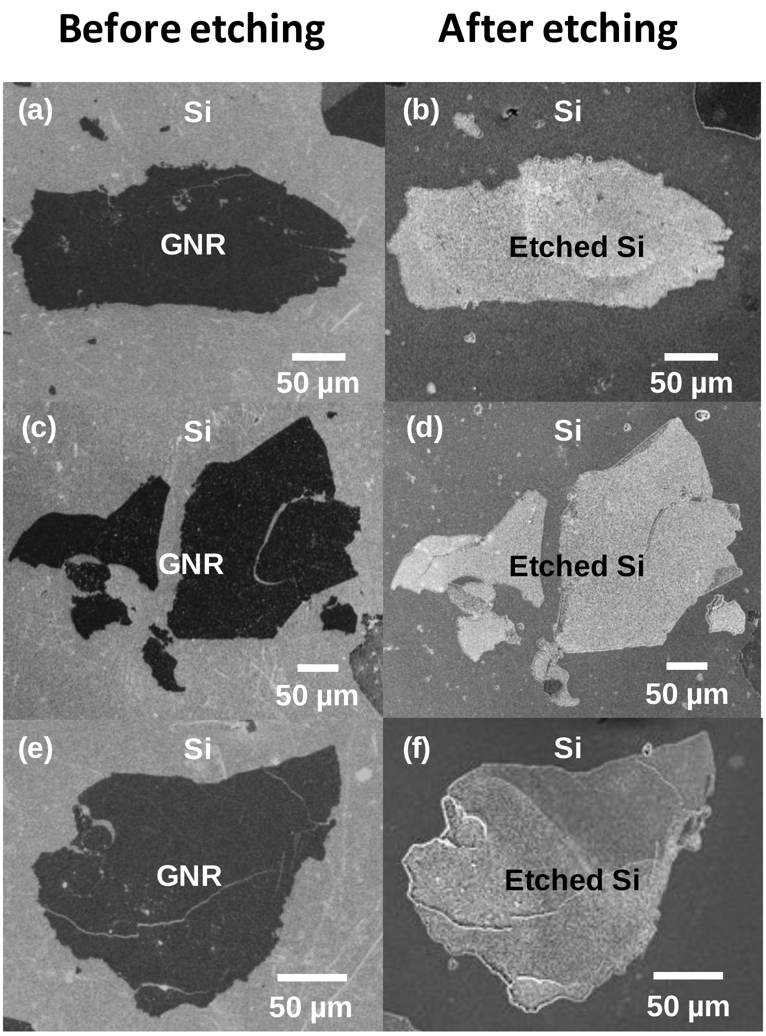

Supplementary Fig. 24. SEM images of GNR-covered Si (a,c and e) before and (b,d and f) after etching.

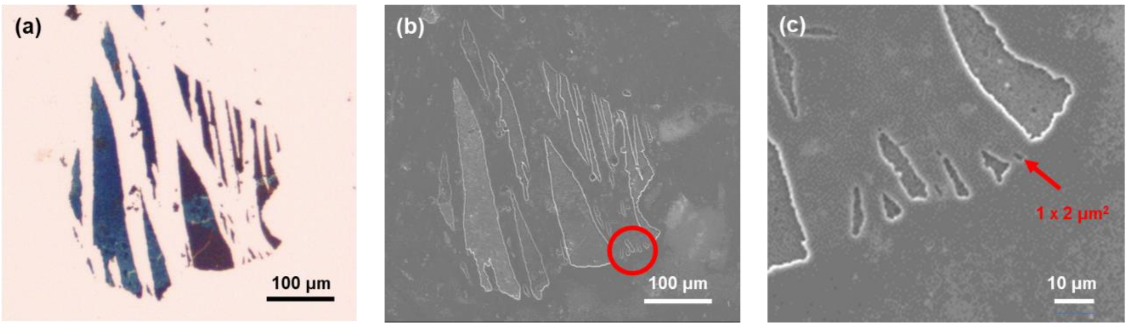

Supplementary Fig. 25. (a) Optical microscope image of GNR-covered Si before etching. (b) SEM image after etching. (c) Magnified SEM image of the red circle in (b).

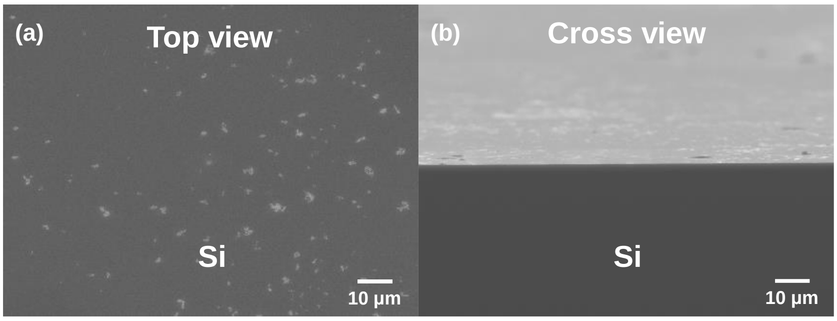

Supplementary Fig. 26. SEM images of top view (a) and cross-sectional view (b) of the 2-butoxynaphthalene precursor-covered Si after etching.

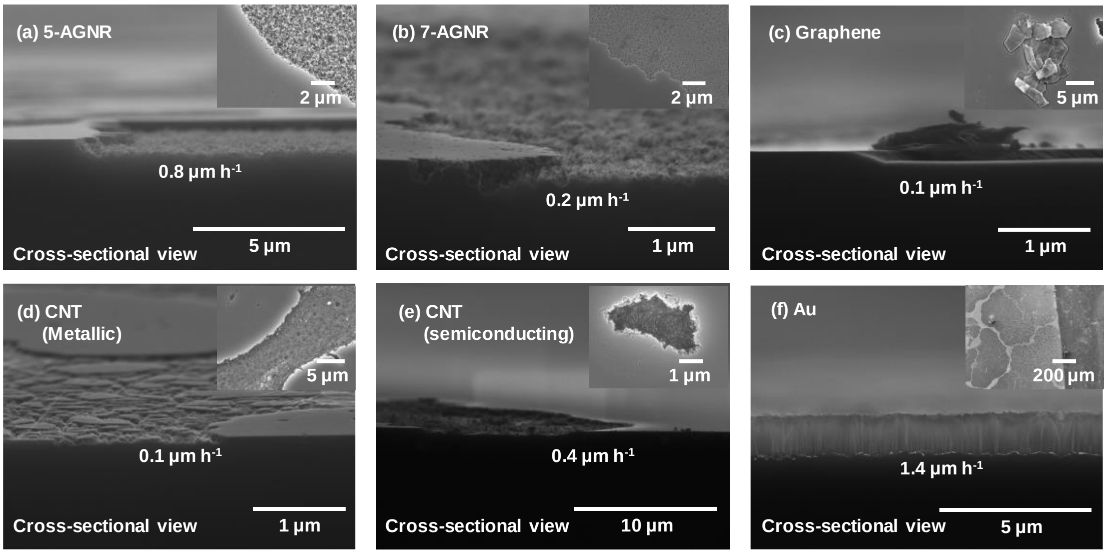

Supplementary Fig. 27. Cross-sectional image of etched-Si substrates catalyzed by (a) 5-AGNR, (b) 7-AGNR, (c) graphene, (d) metallic CNT, (e) semiconducting CNT and (f) gold. The insets show top views.

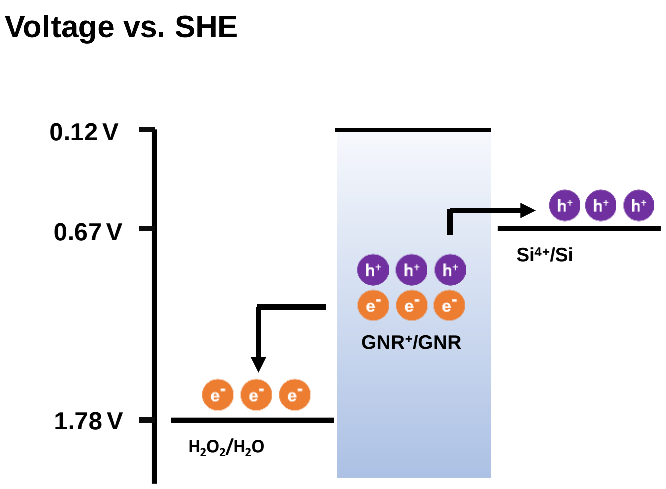

$$H_2O_2 + 2H^+ + 2e^- \rightarrow H_2O$$
$$Si + 6HF + nh^+ \rightarrow H_2SiF_6 + nH^+ + (4 - n)/\,2H_2$$

Supplementary Fig. 28. Illustration of the standard electrode potential of H_2O_2, electrochemically produced GNR and Si to explain the mechanism of vapor-phase chemical etching of Si.

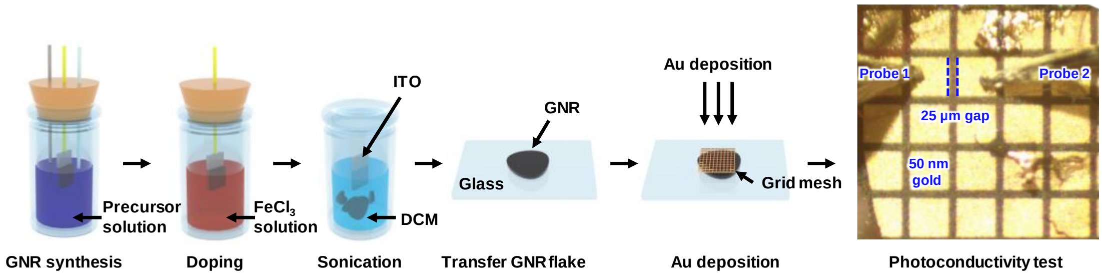

Supplementary Fig. 29. Fabrication of the photoconductive cell of electrochemically produced GNRs.

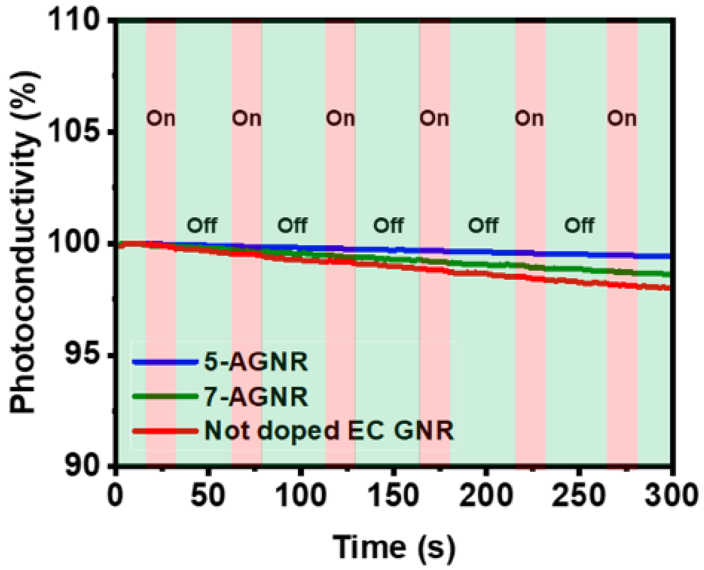

Supplementary Fig. 30. Photoconductive properties of $FeCl_3$-doped 5-AGNR and 7-AGNR and undoped electrochemically produced GNR.

Supplementary References

1. Sakaguchi, H. *et al.* Width-Controlled Sub-Nanometer Graphene Nanoribbon Films Synthesized by Radical-Polymerized Chemical Vapor Deposition. *Adv. Mater.* **26**, 4134–4138 (2014).

2. Clark, S. J. *et al.* First principles methods using CASTEP. *Zeitschrift für Kristallographie - Crystalline Materials* **220**, 567–570 (2005).

3. Perdew, J. P., Burke, K. & Ernzerhof, M. Generalized Gradient Approximation Made Simple. *Phys. Rev. Lett.* **77**, 3865–3868 (1996).
